# Supplementary material for: Hematological Markers in Thromboembolic Events: A Comparative Study of COVID-19 and Non-COVID-19 Hospitalized Patients
Source: J Clin Med. 2025 May 5;14(9):3192. doi: 10.3390/jcm14093192 (PMC12072893; doi:10.3390/jcm14093192)
Supplement: Supplementary file 1 [file jcm-14-03192-s001.zip › Supplementary Table S1 (R1).pdf]

Supplementary Table S1. Sample, mean, confidence interval of renal function parameters in sociodemographic variables in the group of patients diagnosed with COVID-19 No COVID-19

	Creatinine						GFR						ALT						AST						GGT					
	No COVID-19			COVID-19			No COVID-19			COVID-19			No COVID-19			COVID-19			No COVID-19			COVID-19			No COVID-19			COVID-19		
	n (%)	Mean	CI (95%)	n (%)	Mean	CI (95%)	n (%)	Mean	CI (95%)	n (%)	Mean	CI (95%)	n (%)	Mean	CI (95%)	n (%)	Mean	CI (95%)	n (%)	Mean	CI (95%)	n (%)	Mean	CI (95%)	n (%)	Mean	CI (95%)	n (%)	Mean	CI (95%)
**Age**	p<0.001			p<0.001			p<0.001			p<0.001			p <0.001			p=0.360			p=0.029			p=0.942			p<0.001			p=0.281		
0-39	50 (8%)	0.60	0.34-0.85	6 (10%)	0.42	0.32-0.52	50 (8%)	125.58	104.82-146.34	6 (10%)	149.92	128.25-165.60	49 (9%)	37.25	1.06-75.56	6 (10%)	60.80	31.68-153.28	49 (9%)	42.00	7.07-76.93	6 (10%)	36.00	8.67-80.67	49 (9%)	142.00	- 99.47-383.47	6 (10%)	115.20	18.96-249.36
40-64	227 (38%)	1.20	0.68-1.72	29 (46%)	0.63	0.57-0.69	226 (38%)	85.83	73.96-98.69	29 (46%)	111.09	106.56-115.63	218 (38%)	35.93	23.33-48.52	29 (46%)	56.26	33.89-78.63	218 (38%)	51.89	20.49-83.29	29 (46%)	37.74	22.31-53.17	218 (38%)	96.96	43.60-150.33	29 (46%)	160.00	59.26-260.74
65 or more	323 (54%)	1.06	0.82-1.29	28 (45%)	1.12	0.77-1.48	323 (54%)	68.72	61.22-76.22	28 (44%)	69.37	57.11-81.64	304 (53%)	42.02	29.54-54.51	28 (44%)	56.48	20.69-92.27	305 (53%)	45.06	29.69-60.43	28 (44%	36.74	22.22-51.26	303 (53%)	91.39	54.39-128.39	28 (44%)	74.30	46.08-102.53
**Sex**	p<0.001			p=0.196			p=0.315			p=0.408			p=0.528			p=0.775			p=0.360			p=0.903			p=0.436			p=0.674		
Female	232 (39%)	0.98	0.68-1.28	23 (37%)	0.68	0.43-0.93	231 (39%)	75.13	64.07-86.19	23 (37%)	97.58	80.04-115.11	223 (39%)	40.24	26.39-54.08	23 (36%)	72.83	25.52-120.15	223 (39%)	55.42	29.09-81.75	23 (37%)	41.89	22.27-61.51	223 (39%)	114.45	57.97-170.93	23 (37%)	85.50	45.75-125.25
Male	368 (61%)	1.18	0.84-1.51	40 (63%)	0.92	0.68-1.15	368 (61%)	79.34	70.44-88.23	40 (63%)	94.82	83.21-106.43	348 (61%)	39.26	27.98-50.54	40 (64%)	48.06	31.38-64.74	349 (61%)	39.79	27.74-51.39	40 (63%)	34.52	23.49-45.54	347 (61%)	78.93	55.55-102.30	40 (63%)	134.12	63.13-205.11
**Severe COVID-19**	n/a			p=0.094			n/a			p=0.069			n/a			p=0.485			n/a			p=0.939			n/a			p=0.459		
No	600 (100%)	1.08	0.86-1.30	35 (56%)	0.87	0.60-1.14	600 (100%)	77.34	70.45-84.22	35 (56%)	91.02	78.32-103.72	600 (100%)	39.73	31.07-48.38	35 (56%)	46.08	25.75-64.40	600 (100%)	47.21	33.42-61.01	35 (56%)	32.46	20.78-44.14	600 (100%)	95.80	66.73-124.87	35 (56%)	80.38	54.21-106.55
Yes	0 (0%)	n/a	n/a	28 (44%)	0.79	0.56-1.03	0 (0%)	n/a	n/a	28 (44%)	100.75	86.25-115.25	0 (0%)	n/a	n/a	28 (44%)	67.96	34.00-101.92	0 (0%)	n/a	n/a	28 (44%)	41.96	25.98-57.94	0 (0%)	n/a	n/a	28 (44%)	155.00	61.06-248.94
**ICU**	p=0.486			p=0.010			p=0.036			p<0.001			p<0.001			p<0.001			p=0.002			p=0.994			p=0.342			p=0.938		
No	560 (93%)	1.09	0.86-1.31	46 (73%)	0.91	0.68-1.14	559 (93%)	77.10	70.15-84.06	46 (73%)	89.09	78.03-100.16	534 (93%)	39.87	31.11-48.63	46 (73%)	60.42	35.30-85.55	535 (93%)	47.56	33.60-61.51	46 (73%)	35.45	25.62-45.27	533 (93%)	94.73	65.36-124.10	46 (73%)	112.03	70.45-153.61
Yes	40 (7%)	0.89	n/a	17 (27%)	0.61	0.47-0.74	40 (7%)	81.06	n/a	17 (27%)	115.38	100.87-129.89	37 (7%)	52.08	n/a	17 (27%)	46.23	25.78-66.68	37 (7%)	55.47	n/a	17 (27%)	42.00	14.66-69.34	37 (7%)	71.69	n/a	17 (27%)	131.38	26.88-289.65
