# Supplementary material for: Hematological Markers in Thromboembolic Events: A Comparative Study of COVID-19 and Non-COVID-19 Hospitalized Patients
Source: J Clin Med. 2025 May 5;14(9):3192. doi: 10.3390/jcm14093192 (PMC12072893; doi:10.3390/jcm14093192)
Supplement: Supplementary file 1 [file jcm-14-03192-s001.zip › Supplementary Table S2 (R1).pdf]

Supplementary Table S2. Sample, mean, confidence interval of inflammatory parameters in sociodemographic variables in the group of patients who were diagnosed with COVID-19 and no COVID-19.

	LDH					
	No COVID-19			COVID-19		
	n (%)	Mean	CI (95%)	n	Mean	CI (95%)
**Age**	p=0.110			p=0.368		
<39	36 (9%)	685.25	504.71-865.79	6 (10%)	538.80	180.52-897.08
40-64	145 (37%)	667.63	540.68-794.57	28 (48%)	598.13	488.88-707.38
>65	213 (54%)	612.12	523.27-700.98	24 (41%)	639.61	517.62-761.59
**Sex**	p =0.055			p =0.916		
Female	155 (39%)	673.71	576.84-770.58	21 (36%)	586.89	476.73-697.05
Male	239 (61%)	599.05	501.12-696.98	37 (64%)	624.18	521.45-726.92
**Severe COVID-19**	n/a			p =0.115		
No	394 (100%)	634.51	566.52-702.50	31 (53%)	560.12	454.27-665.96
Yes	0 (0%)	n/a	n/a	27 (47%)	663.96	554.67-773.25
**ICU**	`p=0.174			p=0.444		
No	375 (95%)	636.29	567.51-705.07	42 (72%)	618.21	526.70-709.72
Yes	19 (5%)	552.23	n/a	16 (28%)	590.00	448.16-731.84

	Ferritin					
	No COVID-19			COVID-19		
	n	Mean	CI (95%)	n	Mean	CI (95%)
**Age**	p=0.015			p=0.126		
<39	26 (9%)	138.35	94.58-371.28	6 (10%)	346.74	43.68-649.80
40-64	104 (37%)	1064.86	514.71-1615.01	29 (47%)	927.10	557.12-1297.08
>65	151 (54%)	350.61	249.84-451.37	26 (43%)	548.73	303.84-793.62
**Sex**	p =0.000			p =0.018		
Female	131 (47%)	574.70	234.54-914.86	21 (34%)	375.72	261.78-489.66
Male	239 (53%)	586.80	337.06-836.55	40 (66%)	876.21	582.71-1169.71
**Severe COVID-19**	n/a			p =0.004		
No	281 (100%)	581.05	377.70-784.41	33 (54%)	436.79	270.41-603.16
Yes	0 (0%)	n/a	n/a	28 (46%)	972.86	617.07-1328.64
**ICU**	p=0.555			p=0.077		
No	270 (96%)	586.87	381.22-792.51	44 (72%)	627.09	400.80-853.37
Yes	11 (4%)	495.78	n/a	17 (28%)	911.42	436.40-1386.44

	Troponin					
	No COVID-19			COVID-19		
	n	Mean	CI (95%)	n	Mean	CI (95%)
**Age**	p=0.052			p<0.001		
<39	17 (7%)	71.05	99.13-241.23	5 (8%)	10.32	10.44-31.08
40-64	91 (39%)	64.48	8.87-137.83	27 (46%)	48.20	16.97-113.38
>65	124 (53%)	172.01	30.66-313.36	27 (46%)	39.26	18.42-60.10
**Sex**	p =0.040			p =0.424		
Female	97 (42%)	108.05	29.78-186.32	21 (36%)	39.92	6.18-73.65
Male	135 (58%)	151.14	7.56-309.83	38 (64%)	40.75	2.66-84.16
**Severe COVID-19**	n/a			p =0.778		
No	232 (100%)	130.67	41.20-220.14	32 (54%)	47.70	7.48-102.88
Yes	0 (0%)	n/a	n/a	27 (46%)	32.93	7.86-57.99
**ICU**	p<0.001			p=0.104		
No	202 (87%)	132.27	41.70-222.84	44 (75%)	51.78	12.14-91.42
Yes	30 (13%)	2649.97	n/a	17 (25%)	7.36	3.14-11.59

	CRP					
	No COVID-19			COVID-19		
	n	Mean	CI (95%)	n	Mean	CI (95%)
**Age**	p =0.332			p =0.780		
<39	50 (8%)	3.04	1.58-7.66	6 (9%)	4.38	3.23-11.99
40-64	226 (38%)	9.09	5.32-12.86	29 (46%)	6.39	3.27-9.50
>65	320 (54%)	6.74	2.97-10.51	28 (44%)	8.15	4.62-11.68
**Sex**	p =0.097			p =0.554		
Female	230 (39%)	6.02	3.60-8.45	23 (36%)	8.01	4.10-11.93
Male	135 (61%)	8.54	4.01-13.08	40 (64%)	6.43	3.81-9.044
**Severe COVID-19**	n/a			p =0.846		
No	596 (100%)	7.40	4.74-9.95	35 (56%)	8.21	4.70-11.72
Yes	0 (0%)	n/a	n/a	28 (44%)	5.71	3.25-8.18
**ICU**	p=0.084			p=0.699		
No	556 (93%)	7.36	4.72-10.00	46 (73%)	7.42	4.78-10.06
Yes	40 (7%)	2.59	n/a	17 (27%)	5.72	2.13-9.30

	IL-6					
	No COVID-19			COVID-19		
	n	Mean	CI (95%)	n	Mean	CI (95%)
**Age**	p =0.09			p =0.681		
<39	6 (6%)	4.82	0.048-9.59	5 (9%)	299.42	449.50-1048.34
40-64	32 (34%)	88.69	20.39-157.00	26 (46%)	211.53	11.31-411.75
>65	56 (60%)	169.11	30.21-308.00	25 (45%)	211.52	67.24-490.27
**Sex**	p =0.185			p =0.562		
Female	43 (46%)	76.85	25.33-128.36	19 (34%)	138.76	24.44-301.97
Male	51 (54%)	185.24	23.54-346.94	37 (66%)	264.52	35.64-493.40
**Severe COVID-19**	n/a			p =0.077		
No	94 (100%)	133.75	46.36-221.14	29 (52%)	152.36	91.30-396.02
Yes	0 (0%)	n/a	n/a	27 (48%)	290.62	84.12-497.13
**ICU**	p=0.669			p=0.760		
No	91 (97%)	135.07	46.59-223.54	41 (73%)	271.38	64.57-478.19
Yes	3 (3%)	49.74	n/a	15 (27%)	70.35	26.60-167.31
