# Supplementary material for: Hematological Markers in Thromboembolic Events: A Comparative Study of COVID-19 and Non-COVID-19 Hospitalized Patients
Source: J Clin Med. 2025 May 5;14(9):3192. doi: 10.3390/jcm14093192 (PMC12072893; doi:10.3390/jcm14093192)
Supplement: Supplementary file 1 [file jcm-14-03192-s001.zip › Supplementary Table S3 (R1).pdf]

Table S3. Sample mean confidence interval of coagulation parameters in sociodemographic variables in the group of patients who were diagnosed with COVID-19 and no COVID-19.

	Platelets						Fibrinogen						D-dimer					
	No COVID-19			COVID-19			No COVID-19			COVID-19			No COVID-19			COVID-19		
	n (%)	Mean	CI (95%)	n (%)	Mean	CI (95%)	n (%)	Mean	CI (95%)	n (%)	Mean	CI (95%)	n (%)	Mean	CI (95%)	n (%)	Mean	CI (95%)
**Age**	p=0.356			p=0.047			p=0.657			p=0.697			p=0.002			p=0.106		
<39	50 (8%)	296,925.00	163,808.47-430,041.53	6 (10%)	355,000.00	226,055.36-483,944.64	49 (8%)	519.00	321.76-716.24	6 (10%)	571.2	283.57-858.83	39 (10%)	1,184.25	114.64-2,253.86	5 (8%)	3,591.20	1411.29-5,771.11
40-64	227 (38%)	257,007.41	203,683.45-310,331.37	29 (46%)	326,343.48	258,126.25-394,560.70	226 (38%)	719.11	632.96-805.26	29 (46%)	665.39	575.09-755.70	128 (34%)	4,452.22	3,034.51-5,869.93	27 (45%)	3,961.09	1,418.17-6,504.00
>65	323 (54%)	271,120.41	240,078.15-302,162.67	28 (4%)	254,326.09	203,693.69-304,958.48	322 (54%)	618.79	567.68-669.90	28 (44%)	596.61	509.71-683.51	212 (56%)	8,770.47	5,660.61-11,880.33	28 (47%)	11,645.57	1,806.59-21,484.54
**Sex**	p=0.035			p=0.926			p=0.016			p=0.412			p=0.564			p=0.028		
Female	232 (39%)	254,126.32	224,543.12-283709.52	23 (36%)	300,766.67	237,287.90-364,245.43	230 (38%)	616.33	548.84-683.83	23 (36%)	613.67	513.59-713.75	158 (42%)	8,352.58	6,034.90-10,670.26	22 (37%)	13,105.83	401.96-25,809.71
Male	368 (61%)	279,880.95	237,785.16-321976.74	40 (64%)	294,442.42	242,251.57-346,633.28	367 (62%)	676.00	618.15-733.85	40 (64%)	631.39	556.15-706.63	221 (58%)	5,650.05	2,407.03-8,893.06	38 (63%)	4,272.85	2,372.62-6,172.08
**Severe COVID-19**	n/a			p=0.740			n/a			p=0.410			n/a			p=0.467		
No	600 (100%)	267,647.50	241,855.97-293,439.03	35 (56%)	308,746.15	248,871.90-368,620.41	597 (100%)	647.66	603.98-691.34	35 (56%)	654.81	570.26-739.36	379 (100%)	6,933.75	4,926.71-8,940.79	33 (55%)	10,043.88	1,346.35-18,741.42
Yes	0 (0%)	n/a	n/a	28 (44%)	284,120.00	248,871.90-368,620.41	0 (0%)	n/a	n/a	28 (44%)	594.28	510.41-678.15	0 (0%)	n/a	n/a	27 (45%)	4,630.72	2,026.15-7,235.29
**ICU**	p=0.846			p=0.515			p=0.765			p=0.642			p=0.410			p=0.023		
No	560 (93%)	264,081.00	238,964.80-289,197.23	46 (73%)	291,431.58	244,639.84-338,223.32	557 (93%)	646.21	602.06-690.36	46 (73%)	632.39	557.94-706.85	365 (96%)	7,005.82	4,978.01-9,033.64	45 (75%)	9,027.08	3,015.22-15,038.94
Yes	40 (7%)	233,248.56	n/a	17 (27%)	312,000.00	230,773.66-393,226.34	40 (7%)	620.19	n/a	17 (27%)	603.92	520.83-687.01	14 (4%)	2,425.76	n/a	15 (25%)	2,606.15	512.12-4,700.19
